# Disseminated Nocardiosis in a Breast Cancer Patient Caused by Nocardia otitidiscaviarum: A Case Report of Tertiary Centre in Saudi Arabia

**DOI:** 10.7759/cureus.22686

**Published:** 2022-02-28

**Authors:** Sharif Kullab, Salem W Basamad, Mosaad Alnwaisir, Mai Alhowari, Erdogan Nohuz

**Affiliations:** 1 Oncology, College of Medicine, King Saud University, Riyadh, SAU; 2 Emergency Medicine, King Saud University, Riyadh, SAU; 3 Obstetrics and Gynecology, Hospices Civils de Lyon (HCL), Lyon, FRA

**Keywords:** metastatic breast cancer, cerebral nocardiosis, nocardia otitidiscaviarum, disseminated nocardiosis, nocardia

## Abstract

Nocardiosis is a rare opportunistic disease that primarily affects patients with deficient immune systems. *Nocardia otitidiscaviarum* is one of the rare species of Nocardia and it represents less than 3% of all Nocardia cases. Clinical presentation can be varied according to the affected organ. This study describes a case of a breast cancer patient who is immunocompromised due to the chemotherapy. This patient presented with a feature of febrile neutropenia. Investigations of this case led to the diagnosis of *Nocardia otitidiscaviarum. *Treatment of this underlying infection required to hold the chemotherapy for good time and to adapt patient-specific cancer treatment according to the balance between both need of cancer control and infection treatment according to the susceptibility test as in our case.

## Introduction

Nocardia is an aerobic Gram-positive branched bacteria that can be found in organic matters such as decomposing vegetation and soil [1]. It might result in human infections that can be localized or disseminated mainly in immunodeficient patients secondary to systemic diseases or immunosuppressant agents use such as chemotherapy. Nocardia can invade the human body through inhalation or after skin inoculation and proceed to cause corresponding symptoms such as cough, hemoptysis, and skin abscess [2,3].

Nocardia has more than 50 species with variable antimicrobial susceptibility. This signifies the importance of differentiating between its different species. *Nocardia otitidiscaviarum* is one of the less commonly isolated species of Nocardia as compared to other species, and it represents almost less than 3% of all Nocardia cases [4]. Brain abscesses caused by Nocardia are uncommon, accounting for approximately 1-2% of all cerebral abscesses, and the diagnosis of cerebral Nocardia is difficult and requires a high index of suspicion based on clinical, biochemical, and radiological findings [5].

We present a case of metastatic breast cancer patient who is immunocompromised by long-term use of chemotherapy and specific anticancer treatment. She presented initially with features of febrile neutropenia and persistent fever of unknown origin (FUO). Investigations of this clinical case led to the diagnosis of disseminated Nocardia with lung, skin, and brain abscess. Management of such cases was observed with challenges in terms of diagnosis and treatment options. Microbiological confirmation was obtained after doing brain biopsy, as all other septic screening samples were negative. This case report aimed to establish the treatment of this underlying infection required to hold the chemotherapy for good time and to adapt patient-specific cancer treatment according to the balance between both the need for infection treatment and cancer control.

## Case presentation

We present a case of a 36-year-old female patient who is known to have breast cancer stage IV of luminal profile (both estrogen receptor {ER} and progesterone receptor {PR} are positive and Her2-neu is negative) metastatic to lymph nodes, liver, and bone since diagnosis. The patient was following up with oncology services for almost three years and was put on successive lines of specific treatment.

She underwent first thoracic spine fixation for compression fracture of T6 associated with retropulsion and spinal cord impingement, then was started on fulvestrant-based hormonal treatment (HT) associated with goserelin as luteinizing hormone-releasing hormone (LHRH) and palbociclib-based cyclin-dependent kinase (CDK) 4/6 inhibitor and denosumab as an antiosteolytic treatment for her bone metastasis. After almost 17 months of this treatment, she showed disease progression and was then put on capecitabine-based chemotherapy for almost three months as a second-line treatment. Third line treatment upon progression was based on olaparib as poly adenosine diphosphate (ADP) ribose polymerase (PARB) inhibitor as she was harboring BRCA 2 mutation. The fourth line treatment was with chemotherapy (CT) and was based on the combination of paclitaxel and carboplatin on a weekly basis. 

**Figure 1:**
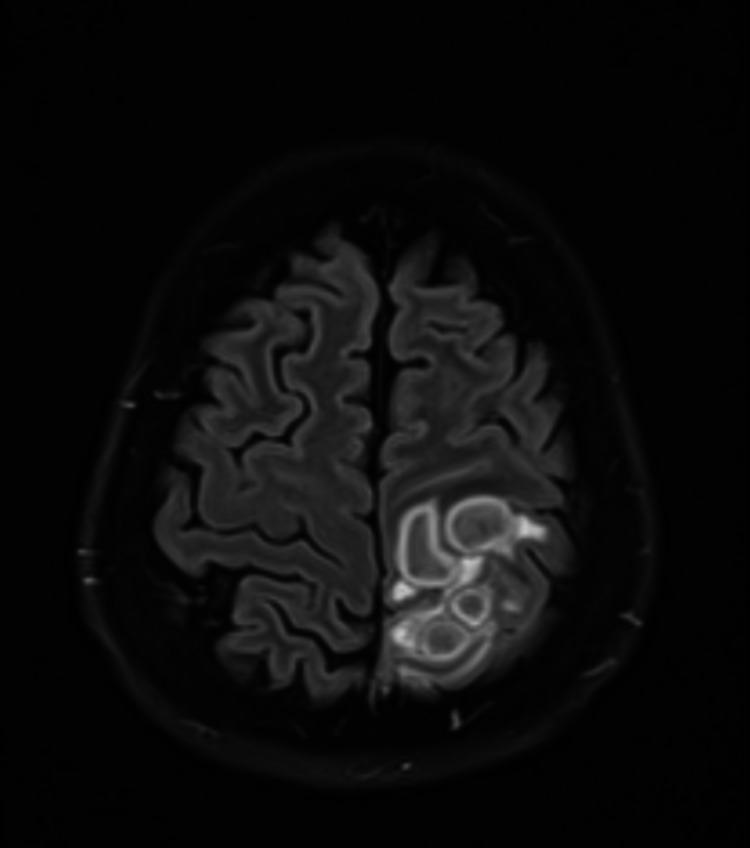
CT brain FLAIR axial view shows multi-locular ring-enhancing lesion in the frontoparietal region surrounding FLAIR hyper-intensity. FLAIR: fluid-attenuated inversion recovery

While the patient was on CT, she presented recurrent episodes of neutropenia that required to postpone her chemotherapy cycles on multiple occasions. She was admitted after three months of CT, with features of febrile neutropenia. Fever was initially considered as fever of unknown origin and was investigated thoroughly. Her chest x-ray was unremarkable. Full septic workup was repeated, and all cultures were negative. CT scan of chest, abdomen, and pelvis was done in order to look for any deep collection, and she was found to have pneumonia. At this stage, she was treated as community-acquired pneumonia (CAP) and was put on piperacillin/tazobactam and vancomycin. However, she did not show clinical or biochemical improvement and started to present multiple subcutaneous small collections and her MRI brain, which was requested to investigate local seizures, showed cerebral abscess (Figure 1). 

In front of this presentation with multiple abscesses (lung, brain, and skin) and in the absence of clinical or biochemical improvement of her symptoms, and failure of isolation of any pathogen in all septic samples, including in cerebrospinal fluid (CSF), a CT guided biopsy from the lung as well as neurosurgical drainage of her brain abscess were carried out. The microbiology result comes back as matrix-assisted laser desorption/ionization-time of flight (MALDI-ToF) positive for *Nocardia otitidiscaviarum. *

Diagnosis of disseminated Nocardiosis was then established almost five weeks after initial admission and investigation of FUO. Thus, the patient’s treatment was adapted according to susceptibility to antibiotics (Table 1). She was finally put on amikacin, linezolid, and ceftriaxone. Then moxifloxacin was added after five weeks.

**Table 1:** Nocardia otitidiscaviarum susceptibility test.

Susceptibility test	
Bactrim	Resistant
Linezolid	Sensitive
Ciprofloxacin	Resistant
Imipenem	Resistant
Moxifloxacin	Intermediate
Cefepime	Resistant
Amoxicillin/clavulanic acid	Resistant
Amikacin	Sensitive
Ceftriaxone	Intermediate
Doxycycline	Intermediate
Minocycline	Intermediate
Tigecycline	Not available
Tobramycin	Resistant
Clarithromycin	Resistant

**Figure 2:**
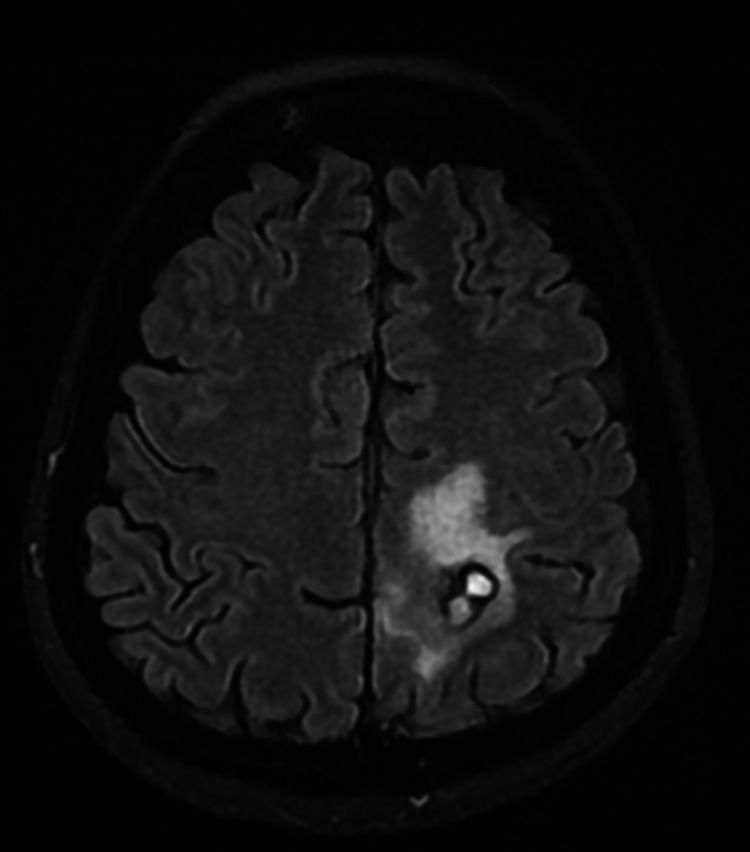
CT brain T2 axial view shows interval regression in the size of the known left partial and posterior frontal abscesses with improvement of the surrounding vasogenic edema.

Since this treatment was started and after nine weeks, we noticed clear clinical, biochemical, and radiological improvement of the patient infection at the different sites (Figure 2). However, in order to avoid dissemination of infection in this immunocompromised patient, decision of holding her chemotherapy was taken by treating medical oncologist during whole this period.

## Discussion

Nocardia is a rare infection that usually infects immunocompromised patients and causes multiple local or disseminated manifestations. It has been identified more than 50 species of Nocardia over the years. *Nocardia otitidiscaviarum* is one of the rare species of Nocardia, and it has been recognized first in 1924 by Snijders in a guinea pig with ear disease [4]. It is rare to find *Nocardia otitidiscaviarum* as the causing organism in patients with nocardiosis-induced infection. A study analysis was done on 374 cases of nocardial infection between 1972 and 1974, and only 10 (2.9%) of them were infected by *Nocardia otitidiscaviarum*, and just one of them had involvement with the central nervous system [4].

In Saudi Arabia, there was a study in 2011 reviewed 30 cases over a period from 1998 to 2006 found that most of the patients had chronic obstructive pulmonary disease (COPD) followed by bronchiectasis. The overall prevalence of Nocardia in Saudi Arabia is not known [5]. Another study overlooked the cases from 2004 to 2018 in a transplantation center and there were 35 cases of Nocardia infection the most common underlying association was transplantation (31.4%) followed by malignancy (20%). They found that pulmonary involvement occurred in 90% of the cases with cough, fever, and dyspnea being the most common symptoms. Nocardia asteroids found to be the main strain isolated in their cases [6]. Nocardia can invade the human body through inhalation of skin insulation and the hematogenously spread to other parts of the body CNS being the most common extrapulmonary site [7].

This bacterium is considered an opportunistic bacterium that usually affects the immunocompromised patient's risk factor include malignancy, HIV infection, and long-term corticosteroids [8]. Our patient has breast cancer stage IV which metastasizes to the lymph nodes, liver, and bone. She received her chemotherapy cycle three months before her presentation.

Nocardia manifestations can present as localized or disseminated with several presentations, the most common is pulmonary nocardiosis [1,9,10]. Pulmonary nocardiosis found to be 83.3% (country) [5] and 71% [11]. The most extrapulmonary site was CNS involvement up to 44%, as in our case, there was multiple brain abscess but no pulmonary involvement and the x-ray was unremarkable at the presentation [7]. Mostly, the clinical symptoms of CNS involvement appear because of brain abscesses causing mass effect and could be mistaken by brain tumor or metastasis, but in our patient, she presented with neutropenic fever that was diagnosed firstly of fever of unknown origin (FUO) [12].

Diagnosis of Nocardia can be missed or delayed since the signs and symptoms of nocardial infection are not specific, diagnosis of Nocardia is challenging, and it needs high index of suspicion. Also, the isolation of Nocardia is difficult due to slow bacterial growth in the laboratory [8,13]. Although the disseminated Nocardia transmits through bloodstream after primary skin or lung infection, Nocardia isolation from blood culture is rare. Some cases have reported positive blood culture for Nocardia sp. [14]. Radiographic finding of pulmonary nocardiosis is non-specific and could be similar to other infections such as tuberculosis and actinomycosis [11]. Diagnosis of Nocardia needs isolation of the organism in clinical specimen. it is important to inform the lab that Nocardia infection is suspected since the Nocardia colonies may take more than two weeks to appear [11]. If the patients have any neurological symptoms, brain imaging should be ordered for these patients [1] and biopsy should be obtained with any positive findings [15].

In the treatment of Nocardia, physicians should start the antimicrobial empirically while awaiting the result of antimicrobial susceptibility test to determine the treatment decision [11]. In the past 50 years, sulfonamides such as sulfadiazine and sulfisoxazole have been the antibiotic of choice to treat nocardial infection [15]. In the United States, trimethoprim-sulfamethoxazole (TMP-SMX) is the most commonly used despite the benefit is unclear [1]. For serious CNS involvement experts recommend three-drug regimens (TMP-SMX + amikacin + either ceftriaxone or imipenem) [11]. Some studies also suggest the addition of linezolid in patient with severe nocardiosis [1]. Therapy of nocardiosis should be until patient improvement occurs. Duration of therapy usually prolonged to minimize the risk of prolapse. Usually treated for six to 12 months of antibiotic therapy [1].

## Conclusions

Nocardia infection is a rare infection but increases along with the number of patients who receive immunosuppressive therapies for hematology or a solid tumor. Pulmonary pneumonia is still kept as a differential diagnosis from pneumonia for other reasons. However, a combination of pneumonia, brain abscess, and subcutaneous abscess needs attention to such infection, especially in immunocompromised patients.

Microbiological diagnosis is difficult in some cases and not easy to obtain without soft tissue biopsy from the affected organ, which might delay the initiation of specific antibiotics. Nocardia can produce serious complications, but early diagnosis and early treatment initiation can lead to successful results, especially in cancer patients. Reasonable control of this condition will help resume specific cancer treatment once the infection is well controlled. This early diagnosis and early specific antibiotics initiation need to be thought about in cancer patients, especially with characteristic clinical and radiological features in immunocompromised patients.
